# Anti-Human Platelet Antigen-1a Immunoglobulin G Preparation Intended to Prevent Fetal and Neonatal Alloimmune Thrombocytopenia

**DOI:** 10.1371/journal.pone.0162973

**Published:** 2016-09-14

**Authors:** Ying-Jan Weng, Anne Husebekk, Björn Skogen, Mette Kjaer, Liang-Tzung Lin, Thierry Burnouf

**Affiliations:** 1 Graduate Institute of Biomedical Materials and Tissue Engineering, College of Biomedical Engineering, Taipei Medical University, Taipei, Taiwan; 2 Department of Medical Biology, UiT The Arctic University of Norway, Tromsø, Norway; 3 Department of Laboratory Medicine, University Hospital North Norway, Tromsø, Norway; 4 Finnmark Hospital Trust, Hammerfest, Norway; 5 Graduate Institute of Medical Sciences, College of Medicine, Taipei Medical University, Taipei, Taiwan; 6 Department of Microbiology and Immunology, School of Medicine, College of Medicine, Taipei Medical University, Taipei, Taiwan; Royal College of Surgeons in Ireland, IRELAND

## Abstract

Fetal and neonatal alloimmune thrombocytopenia (FNAIT) is a severe disease that is caused by maternal alloantibodies generated during pregnancy or at delivery as a result of incompatibility between maternal and fetal human platelet antigens (HPAs) inherited from the father. Antibody-mediated immune suppression using anti-HPA-1a immunoglobulins is thought to be able to prevent FNAIT caused by HPA-1a. A fractionation process to prepare anti-HPA-1a immunoglobulin (Ig) G (IgG) from human plasma was therefore developed. Anti-HPA-1a plasma was obtained from volunteer mothers who underwent alloimmunization against HPA-1a during a previous pregnancy. Plasma was cryoprecipitated and the supernatant treated with caprylic acid and solvent/detergent (S/D), purified by chromatography, nanofiltered, concentrated, and sterile-filtered. The anti-HPA-1a immunoglobulin fraction was characterized for purity and safety. PAK12 and quantitative monoclonal antibody immobilization of platelet antigen (MAIPA) assays were used to detect anti-HPA-1a IgG. Hepatitis C virus (HCV) removal during nanofiltration was assessed by spiking experiments, using cell culture-derived reporter HCV and luciferase analysis. The caprylic acid treatment precipitated non-Ig proteins yielding a 90% pure Ig supernatant. S-HyperCel chromatography of the S/D-treated supernatant followed by HyperCel STAR AX provided high IgG recovery (>80%) and purity (>99.5%), and efficient IgA and IgM removal. Concentrations of complement factors C3 and C4 were < 0.5 and < 0.4 mg/dL, respectively. The final IgG could be nanofiltered on Planova 20N under conditions removing more than 3 log HCV infectivity to baseline mock infection level, and concentrated to ca. 30 g/L. Proteolytic activity and thrombin generation were low in the final fraction. The Pak12 and MAIPA assays showed good recovery of anti-HPA-1a throughout the process. Clinical-grade HPA-1a IgG can be prepared using a process compliant with current quality requirements opening perspectives for the prevention of FNAIT.

## Introduction

Fetal and neonatal alloimmune thrombocytopenia (FNAIT) is caused by the generation of maternal alloantibodies as a result of incompatibility between maternal and fetal human platelet antigens (HPAs) inherited from the father [1,2]. FNAIT occurs in about 40 per 100’000 pregnancies, and the most feared complication, intracranial bleeding (ICH) in fetuses and newborns, in 3 or 4 children per 100’000 [3,4]. ICH may result in severe neurologic sequelae, miscarriage, and neonatal death [5]. Maternal immunization may take place during pregnancy or at delivery, exerting a potential impact on the present and subsequent incompatible pregnancies [1,6]. In Caucasian populations, HPA-1a is an antigen located on the extracellular part of the β3 integrin subunit (GPIIIa) on αIIbβ3 (GPIIbIIIa). No screening for FNAIT is performed and mothers with affected children are likely to be treated with intravenous immunoglobulin (IVIG), with or without steroids, in any subsequent pregnancies [7,8]. Intrauterin platelet transfusions are not recommended due to high risk of bleeding complications and the efficacy of this invasive fetal platelet transfusion has not been well studied [9].

There is currently no established specific treatment for prevention of maternal immunization. However, the pathophysiology of FNAIT appears similar to that of the hemolytic disease of fetuses and newborns (HDFN), in which alloimmunization induced by the RhD antigen on red blood cells takes place late in the pregnancy, or at the time of delivery following a small feto-maternal hemorrhage [10–14]. Alloimmunization against the RhD antigen can efficiently be prevented through antibody-mediated immune suppression (AMIS) by the passive administration of plasma-derived anti-D immunoglobulin (Ig) G [15], a preparation that is listed on the World Health Organization Model List of Essential Medicine. It has now been suggested that AMIS using IgG directed against HPA-1a can also be a prophylactic strategy to prevent maternal alloimmunization and FNAIT [16]. A pre-clinical demonstration of the rationale of this approach was obtained in a murine model where injection of an experimental plasma-derived anti-HPA-1a IgG purified by protein G chromatography prevented FNAIT [17]. In this study, we have now looked, as a proof of concept, at the possibility of preparing clinical-grade plasma-derived anti-HPA-1a IgG using a fractionation process meeting current regulatory requirements for optimal product purity and safety [18]. Developing a dedicated purification process of small volumes of anti-HPA-1a plasma is justified as the current plasma fractionation technology using ethanol fractionation is designed for processing very large plasma pools (e.g. 4000 liters) [18] and does not provide optimal recovery of IgG [19].

## Materials and Methods

### Plasma samples collection

Anti-HPA-1a-positive plasma was collected by apheresis from four Norwegian women (approximately 500 mL per donor) who provided written informed consent. These consenting donors developed alloimmunization against HPA-1a during a previous pregnancy. and delivered severely thrombocytopenic children. Collected plasma was frozen and stored at -80°C until use. The ethics committee of the North Norway University approved the study.

### Preparation of the anti-HPA-1a IgG fraction

Experiments were initially done using 50 mL of plasma. IgG was purified largely as described previously [20], with modifications to incorporate viral-reduction steps. Plasma was thawed at 2~4°C overnight to form a cryoprecipitate slurry that was removed by centrifugation at 6000 *x*g for 30 min at 4°C (Beckman Avant J-25 Centrifuge; Beckman Coulter, Brea, CA, USA). The supernatant (cryoprecipitate (cryo)-poor plasma; CPP) was subjected to a 5% (v/v) caprylic acid (Sigma, St. Louis, MO, USA)/pH 5.5 precipitation step at 20~25°C under constant mild stirring for 60 min [20,21]. The precipitate was removed by centrifugation at 6000 *x*g for 30 min at 25°C. The cryoprecipitate-poor supernatant was adjusted to pH 4.5 and a conductivity of 8 mS/cm using 25 mM sodium acetate buffer, and subjected to solvent/detergent (S/D) treatment using 1% (v/v) tri-n-butyl phosphate (Merck, Darmstadt, Germany) and 1% (v/v) Triton X-100 (Sigma) for 1 h at 25 ± 2°C. The plasma fraction was then purified using 5 mL of S-HyperCel cation exchanger (Pall Life Sciences, Cergy, France) equilibrated in 25 mM acetate buffer, at a load of 30 mg protein/mL and at a 298-cm/h linear flow rate. Following injection, the column was washed with 10 column volumes of equilibration buffer to remove unbound proteins and eliminate the S/D agents. The IgG fraction was eluted by 25 mM Tris-HCl at pH 8.0 and 12 mS/cm and directly injected onto a 1-mL HyperCel STAR AX anion exchanger (Pall Life Sciences) equilibrated in the same buffer, at a 153-cm/h linear flow rate [20]. The purified IgG recovered in the column breakthrough (ca. 30 mL) was filtered on 0.2 μm and subjected to 0.01 m^2^ Planova 20N (Asahi-Kasei Medical, Tokyo, Japan) virus removal nanofiltration at 35 ± 0.5°C and a transmembrane pressure of < 1 bar. The nanofiltered IgG fraction (ca. 27 mL) was concentrated about 10 times using a centrifugal device (GE, Sartorius, Göttingen, Germany) and then dialyzed against phosphate-buffered saline (PBS) in a dynamic dialysis device (Spectrum Lab, Rancho Dominguez, CA, USA). Samples were taken throughout the purification process and stored at -80°C until analysis. For scale-up experiments, plasma, S-HyperCel, and HyperCel STAR AX volumes were 250, 25, and 5 mL, respectively, and the same processing parameters were used.

### Immunological and physicochemical assays

The total protein concentration was determined by the Bradford method (Coomassie Plus Protein Assay Kit, Thermo, Waltham, MA, USA), and IgG, IgA, and IgM concentrations by sandwich enzyme-linked immunosorbent assay (ELISA) kits (Human IgG, IgA, and IgM ELISA Quantitation Set, Bethyl Laboratories, Montgomery, TX, USA) [20]. Complements C3 and C4 were measured with a polyethylene glycol (PEG)-enhanced immunoturbidimetric method using an ADVIA 2400 Chemistry System analyzer (Siemens Healthcare Diagnostics, city?, NY, USA). Albumin determination used a bromocresol green solution (BCG) as a binding dye, and the albumin-BCG complex was measured as an endpoint reaction at 596/694 nm. The purity of the gamma protein was assessed by zone electrophoresis using a Hydragel 7 protein kit (Sebia, Evry, France) [22]. Sodium dodecylsulfate polyacrylamide gel electrophoresis (SDS-PAGE) under non-reducing or reducing conditions was run using a NuPAGE 4%~12% Bis-Tris Gel (Invitrogen, Carlsbad, CA, USA) and a PageRuler prestained protein ladder (Thermo). Separated proteins were stained with Protein Gel Fast Stain (Strong Biotech, Taipei, Taiwan) [22].

### Thrombin generation and proteolytic activity assays

Thrombin generation was assessed as described before [21,22] with a Technothrombin thrombin generation assay (TGA) kit (Technoclone, Vienna, Austria) using the RC High reagent. CPP and the final IgG fraction were used to spike platelet-poor plasma (PPP) at 10% and 2% (v/v), respectively, to ensure similar final IgG concentrations. Proteolytic activity was assessed by chromogenic assays to detect plasmin (S-2251), thrombin-specific activity (S-2238), and thrombin-like proteolytic activity (S-2288) using a chromogenic substrate (Chromogenix, Milan, Italy), and results were expressed in units of activity/g protein (U/g), as described before [21,22].

### Detection of HPA-specific antibodies

The presence of anti-HPA-1a was checked by a qualitative/semiquantitative solid-phase ELISA using Lifecodes PAK 12 (Immucor GTI Diagnostics, Waukesha, WI, USA), an assay that detects antibodies to HLA class I antigens and to epitopes on the platelet glycoproteins IIb/IIIa, Ia/IIa, and Ib/IX. Undiluted plasma fractions and control samples at 50 μL each were added to microwells and incubated for 30~35 min at 37°C. Unbound antibodies were then washed away, and 50 μL of diluted alkaline phosphatase-labeled anti-human globulin reagent (anti-IgG/A/M) was added to the wells and incubated. Unbound anti-IgG/A/M was washed away, and 100 μL of p-nitrophenyl phosphate (PNPP) substrate was added and incubated for 30 min before stopping the reaction with 100 μL of stopping solution. The absorbance (OD) of each well was measured at 405 or 410 nm on an ELISA reader (MULTISKAN GO, Thermo) using a reference filter of 490 nm. More-precise quantitation of the anti-HPA-1a level in fractions was determined by a monoclonal antibody immobilization of platelet antigen (MAIPA) assay [23] performed at the University of Tromsø The Arctic University of Norway [24] by a method that was slightly modified from that described by Kiefel et al. [25]. A standard curve was made with dilution of plasma from an alloimmunized woman.

### Hepatitis C virus (HCV) removal by Planova 20N nanofiltration

The methodology to obtain and assess cell culture-derived HCV (HCVcc) particles titers and sample cytotoxicity was described before [26]. HCVcc particles were produced by electroporation of hepatoma Huh-7.5 cells using the *Gaussia* luciferase reporter-tagged Jc1FLAG2(p7-nsGluc2A) construct (genotype 2a; kindly provided by Dr. Charles M. Rice) [27]. HCV viral titers were determined as the 50% tissue culture infectious dose (TCID_50_) [28] using immunofluorescence staining with NS5A. The cytotoxicity of HyperCel STAR AX IgG samples was evaluated against Huh-7.5 cells prior to all experiments. Cells were cultured in Dulbecco's modified Eagle medium (DMEM) supplemented with 10% fetal bovine serum (FBS), 50 μg/ml gentamicin, and 0.5 μg/ml amphotericin B. Subconfluent Huh-7.5 cells (10^4^ cells/well of 96-well plates) were treated with purified IgG samples with or without dilution (1:10 in PBS), and cell viability (%) was assessed at day 3 or 5 post-treatment using a Cell Counting Kit (CCK)-8 (Sigma). For viral-removal studies, IgG samples (20 mL) were spiked with the HCVcc stock at a ratio of 90:10 (IgG:HCV stock) [29]. HCV-spiked IgG samples were passed through a 0.001-m^2^ Planova 20N filter. Samples containing IgG only, HCV-spiked IgG, or HCV-spiked IgG nanofiltered on Planova 20N were then evaluated for viral infectivity. Huh-7.5 cell monolayers (2 × 10^5^ cells/well of 12-well plates) were challenged at 37°C for 3 h with IgG samples followed by removal of any excess virion by two PBS washes before incubation in medium containing 2% FBS. The supernatant was collected on day 4 post-infection and then assayed for luciferase activity using the BioLux Gaussia Luciferase Assay Kit (New England Biolabs, Pickering, ON, Canada) and a luminometer (Promega, Madison, WI, USA). HCV infectivity was expressed as log_10_ of relative light units (RLU) as previously reported [26].

### Statistical analysis

Data shown were collected from at least three independent experiments and are presented as the mean ± standard deviation (SD).

## Results

### Fractionation scheme

**Fig 1:**
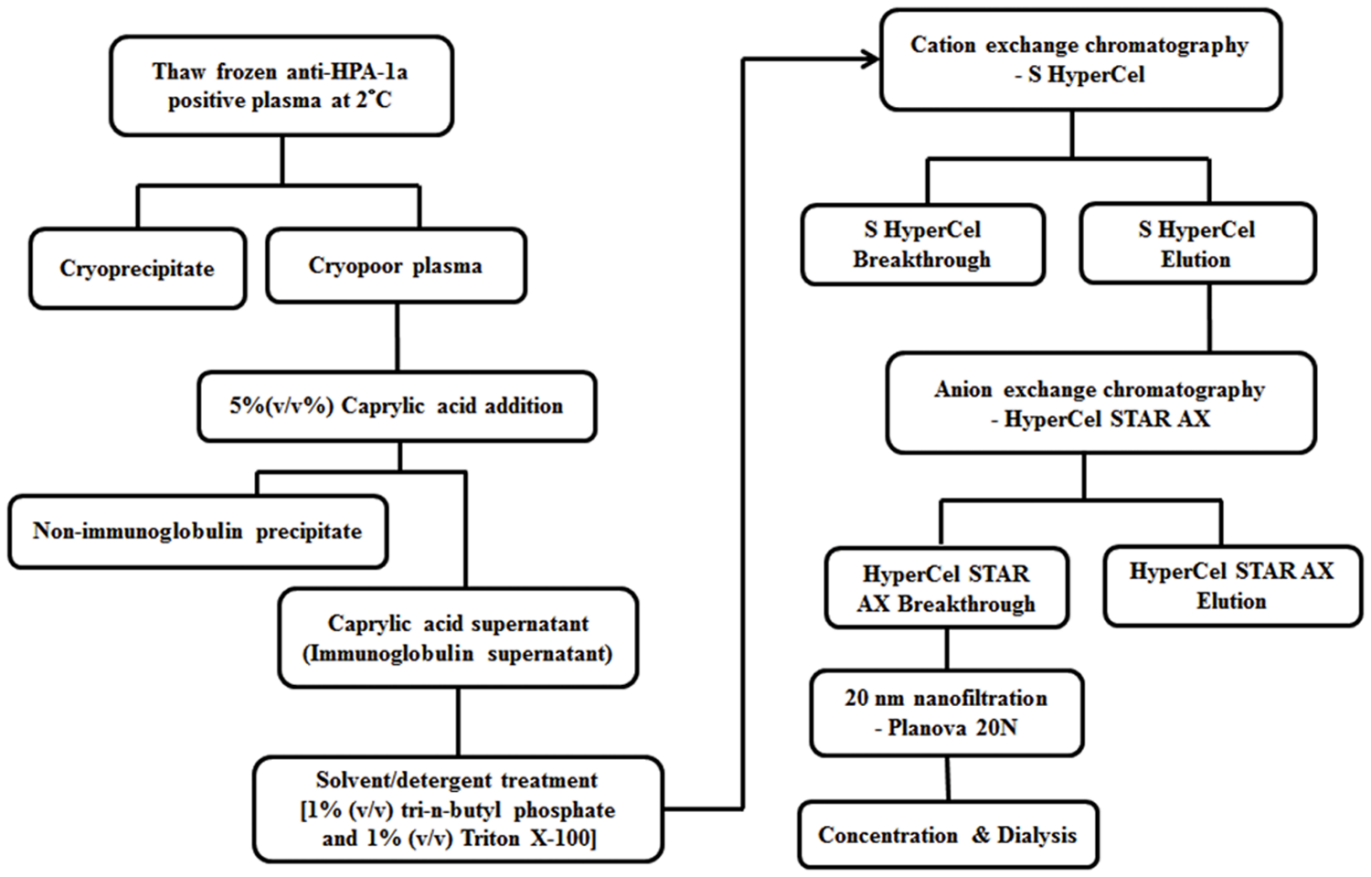
Fractionation scheme of plasma for the preparation of anti-human platelet antigen (HPA)-1a immunoglobulin G.

Characteristics of the content in anti-HPA-1a IgG in the four starting plasma donations and information on the respective plasma donors (severity of FNAIT, occurrence of ICH, and anti-HPA-1a IgG content by MAIPA at different time points) are presented in Table 1. The IgG fractionation process applied in this study is summarized in Fig 1.

**Table 1:** Characteristics of plasma donation and alloimmunized plasma donors.

		Donor number			
		1	2	3	4
**Plasma donation used in the study**					
PAK-12 assay, OD*	1a, 3a, 4a	NA	2.33	1.60	1.72
	1b, 3b, 4a	NA	0,18	0,11	0,10
	1a, 3b, 4a	NA	2,56	1,58	1,84
MAIPA, IU/mL		129	127	27	33
**Plasma donor data**					
FNAIT severity		Delivery of twins with platelet count of 237 and 295 x 10^3^/μL, respectively	NA**	NA**	Delivery of baby with Platelet count of 100 x 10^3^/μL
ICH occurrence		No	NA**	NA**	No
MAIPA, IU/mL	At delivery (year)	1.99 (2003)	277.5 (2002)	19.8 (2006)	6.8 (2000)
	After delivery (year)	189.6 (2009)	202.8 (2009)	42.4 (2009)	62.8 (2009)

* optical density

**NA: not available (identification of alloimmunization at the blood bank, not in association with a delivery)

**Fig 2:**
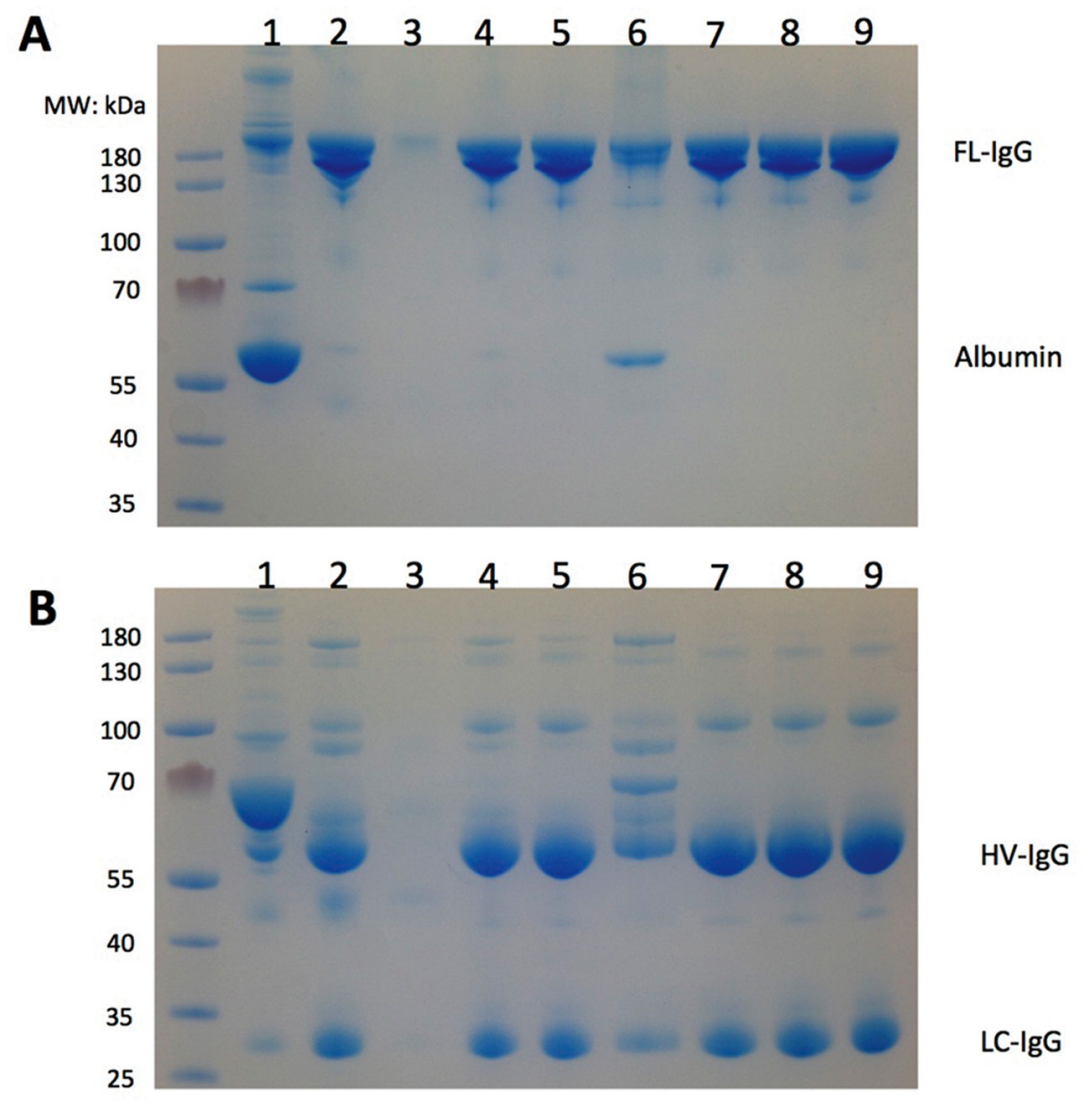
SDS-PAGE profile of anti-human platelet antigen (HPA)-1a fractions generated throughout the purification process. A: Non-reducing conditions; B: reducing conditions. 1: cryoprecipitate-poor plasma; 2: caprylic acid supernatant; 3: S HyperCel breakthrough; 4: S HyperCel eluate; 5: HyperCel STAR-AX breakthrough; 6: HyperCel STAR-AX eluate; 7: after nanofiltration; 8: after concentration; 9: final fraction after dialysis. The left lane shows molecular weight markers. Staining was with Coomassie blue. FL-IgG, intact whole IgG; HC-IgG, heavy-chain IgG; LC-IgG, light-chain IgG.

Briefly anti-HPA-1a IgG was first purified by an upstream process comprising cryoprecipitation, followed by caprylic acid precipitation of non-Ig proteins. The IgG fraction was then polished by on-line cation-exchange and anion-exchange chromatography (Fig 2). Two dedicated viral-reduction steps were integrated in the purification process: S/D treatment and 20-nm nanofiltration. The recovery of IgG was more than 60%~65% from CPP and was 80%~85% from the caprylic acid supernatant.

### Analysis of in-process intermediates

**Fig 3:**
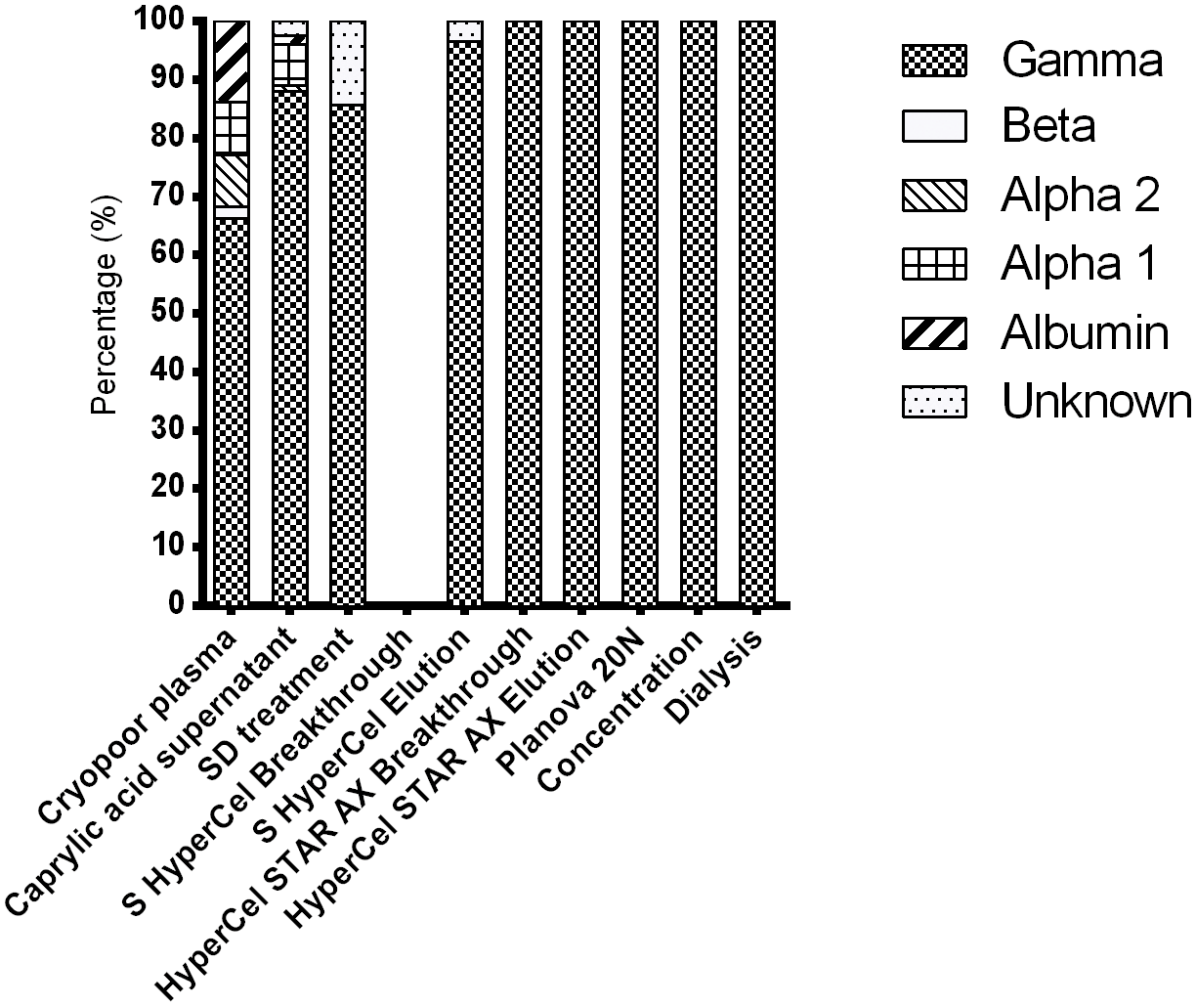
Densitometric analysis of the zone electrophoresis of fractions generated throughout the purification process showing the relative proportions of proteins migrating as gamma, beta, alpha-2, and alpha-1 proteins, and albumin.

Fig 2A shows the SDS-PAGE protein profile (under non-reducing conditions) of various fractions along the purification process, revealing a gradual increase in IgG purity. The CPP pattern (lane 1) was characterized by two major protein bands corresponding to albumin (ca. 67 kDa) and IgG (ca. 150 kDa). Caprylic acid treatment removed most proteins apart from IgG (lane 2). The S-HyperCel breakthrough (lane 3) contained minor protein contaminants and essentially no IgG. The S-Hypercel eluate (lane 4) and the HyperCel STAR AX breakthrough (lane 5) contained proteins at a MW migration expected for IgG. The HyperCel STAR AX eluate (lane 6; discarded fraction) contained proteins with a molecular weight (MW) of albumin and some Igs. The protein profile did not change during nanofiltration (lane 7), concentration (lane 8), or dialysis (lane 9). The electrophoretic profile under reducing conditions (Fig 3B) allowed detection of two protein bands with MWs of 50 and 25 kDa corresponding to IgG heavy and light chains, respectively.

Zone electrophoresis (Fig 3) confirmed the increment in IgG purity along the process, and the final fraction after HyperCel STAR AX contained 100% of proteins migrating in the gamma zone.

**Fig 4:**
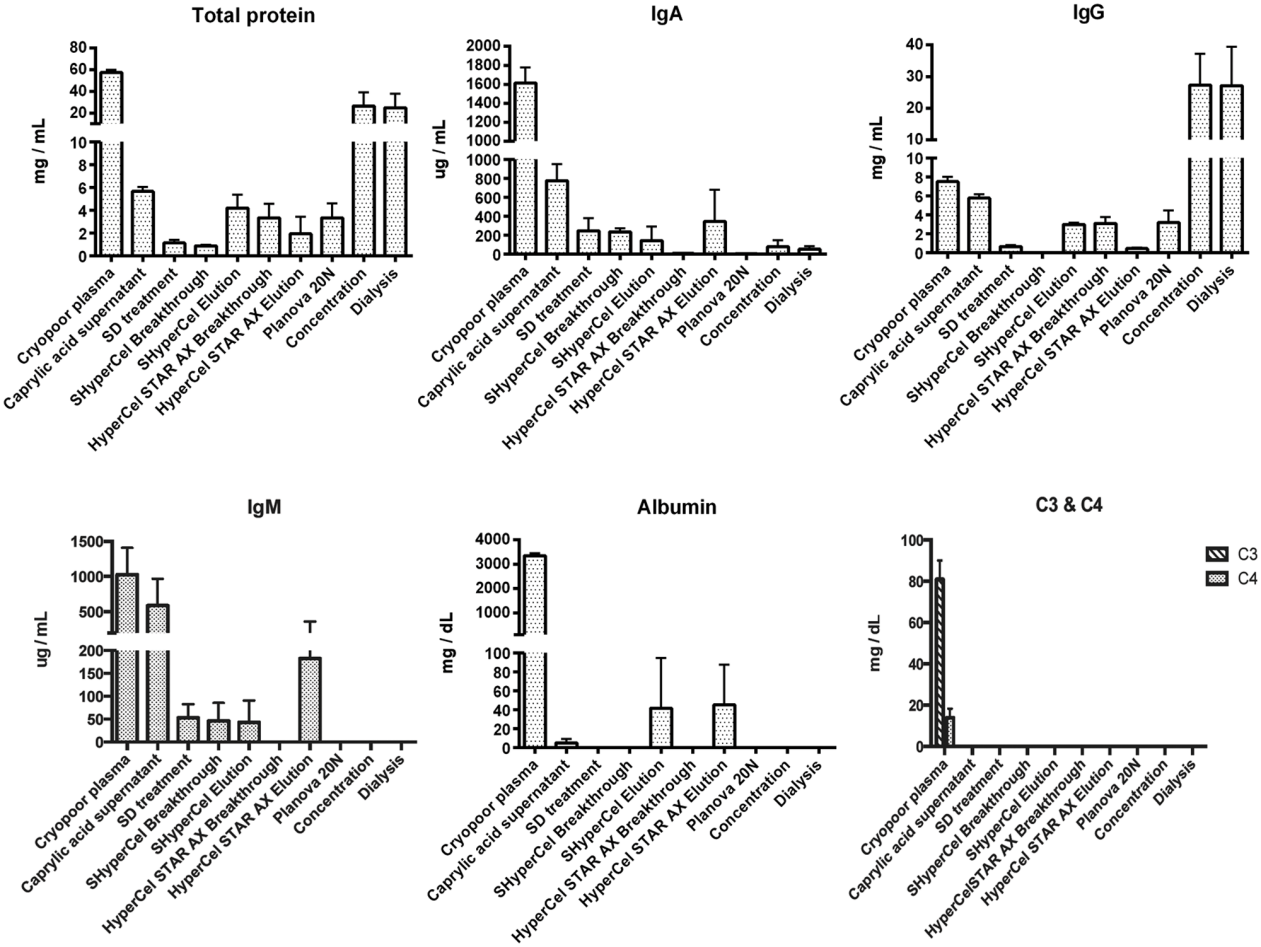
Contents of total proteins, immunoglobulin G (IgG), IgA, IgM, albumin, and complement 3 (C3) and C4 fractions throughout the purification process.

Fig 4 presents contents of total proteins, IgG, IgA, IgM, albumin, and C3/C4 complement components. The purified fraction had a mean IgG content of 27.1 ± 12.3 mg/mL and a purity of >99.5%, and was essentially free of IgA (< 0.05 mg/mL), IgM (< 0.015 mg/mL), albumin (< 0.017 mg/mL), and complement components C3(< 0.005 mg/mL) and C4 (< 0.004 mg/mL). Most of the IgA and IgM present in the caprylic acid supernatant was removed by the two chromatographic steps, and residual albumin was removed by HyperCel STAR AX. C3 and C4 were removed by caprylic acid precipitation.

The TGA data using the RC high reagent (Table 2) showed only a low thrombin generation capacity in CPP (151.57 ± 12.38 nM/L) as well as in final IgG (154.59 ± 27.20 nM/L). Thrombin (undetectable), proteolytic activity (0.02 U/g), and plasmin (undetectable) were much less than in the starting CPP (1.71, 0.31, and 5.52 U/g, respectively) showing the contribution of the developed process to remove corresponding proteases.

**Table 2:** Thrombin generation assay using RC high comparing cryoprecipitate-poor plasma and purified anti-HPA-1a IgG. Mean +/- SD (mini-maxi). AUC: area under the curve.

	RC High				
	Lag phase, min	Thrombin, nmol/L	Time to peak, min	Velocity index	AUC
Cryo-poor plasma	10.3±0.6 (10–11)	151.6±12.4 (139.5–164.2)	22.0±1.0 (21–23)	13.05±1.7 (11.6–14.9)	2590.1±107.4 (2502–2710)
Final IgG	10.1±1.5 (7–12)	154.6±27.1 (120.4–207.5)	22.4±3.8 (15–28)	13.6±5.6 (8.4–25.9)	2695.1±145.3 (2466.2–2862.4)

**Fig 5:**
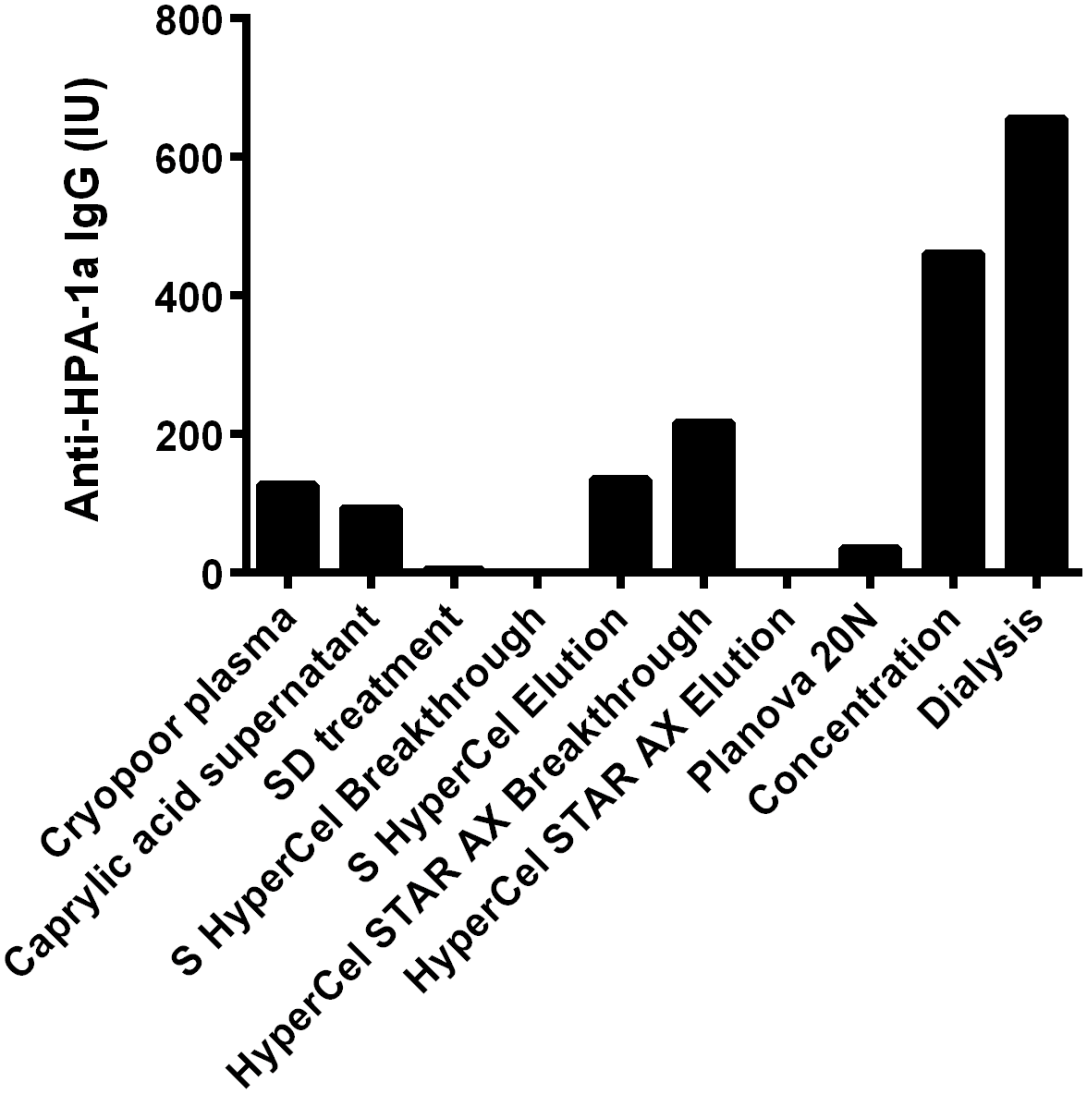
Quantitative monoclonal antibody immobilization of platelet antigen (MAIPA) assay of anti-HPA-1a IgG at different step of the purification process.

The PAK-12 semiquantitative assay (Table 3) detected the presence of anti-HPA-1a IgG in CPP, the caprylic acid supernatant, S/D-treated fraction, and S-Hypercel elution, HyperCel STAR AX breakthrough, Planova 20N, dialysis and concentration steps. The MAIPA assay confirmed these data. In a typical 250-mL batch, the anti-HPA-1a content was about 120 IU/mL in CPP, 100 IU/mL in the caprylic acid supernatant, 180 IU/mL in the S HyperCel eluate and HyperCel STAR-AX breakthrough, and 665 IU/mL in the nanofiltered, concentrated, and dialyzed product (Fig 5).

**Table 3:** Semi-quantitative determination of platelet antigens by PAK-12 assay.

Platelet antigens	Cryo-poor plasma	CA supernatant	S/D treatment	S HyperCel		HyperCel STAR AX		Planova 20N	Concentration	Dialysis
				Breakthrough	Eluate	Breakthrough	Eluate			
**1a, 3a, 4a**	**2.05**	**1.57**	**0.62**	0.08	**2.55**	**2.56**	0.23	**2.48**	**2.43**	**2.47**
**1b, 3b, 4a**	0.14	0.16	**0.50**	0.09	0.06	0.06	0.06	0.05	0.24	0.34
**1a, 3b, 4a**	**1.99**	**1.58**	**0.56**	0.09	**2.45**	**2.45**	0.22	2.50	**2.33**	**2.29**

### HCV is removed by nanofiltration on Planova 20N

**Fig 6:**
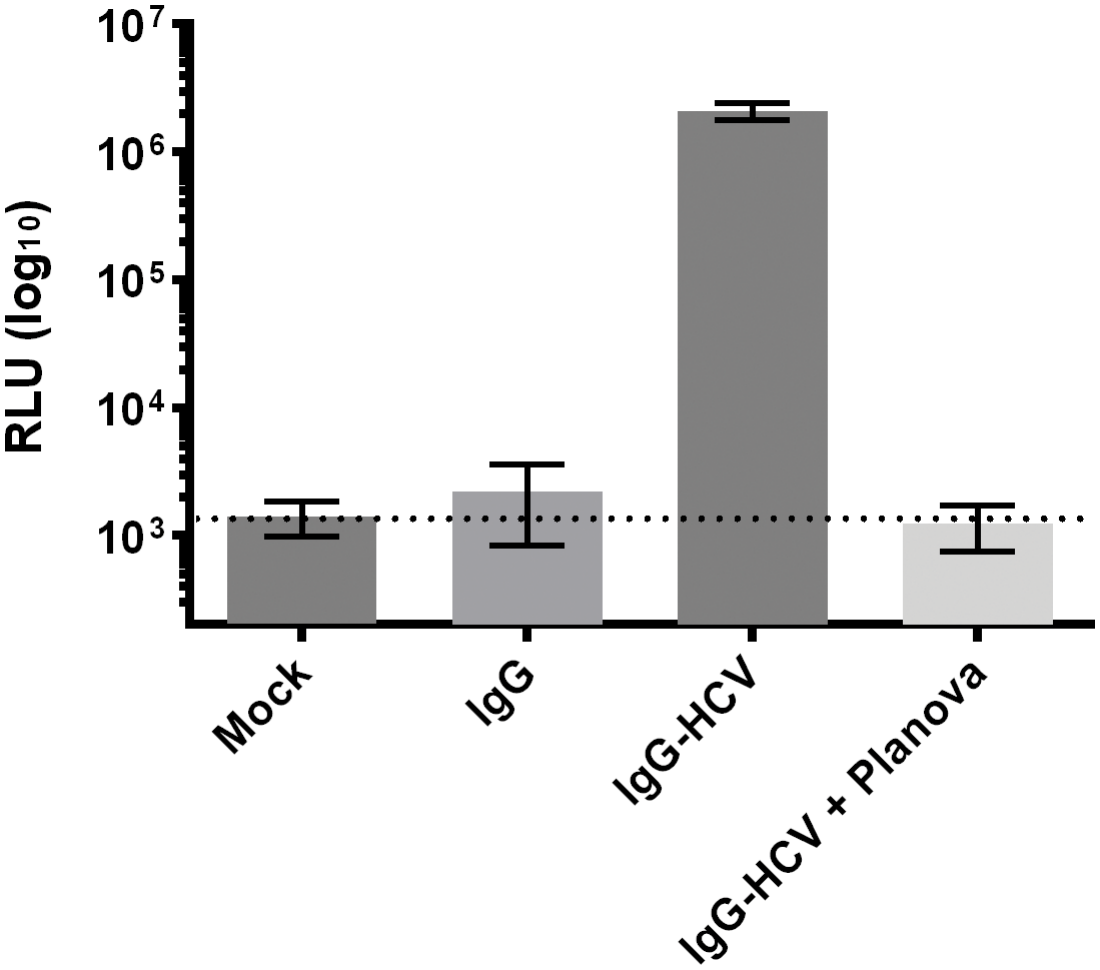
Removal of the hepatitis C virus (HCV) during nanofiltration as determined using luciferase reporter measurements (log_10_ RLU). *N* = 3. Mean ± SEM. The dashed line indicates the background level of luciferase activity.

Cell viability was first determined to verify that the purified undiluted anti-HPA-1a IgG fraction obtained after HyperCel STAR AX breakthrough was not cytotoxic to Huh-7.5 cells. Similarly, we detected no toxicity of IgG after nanofiltration. To further analyze the capacity of Planova 20N to remove HCV infectivity, 20 mL of HCV-spiked anti-HPA-1a IgG was subjected to nanofiltration on a 0.001-m^2^ Planova 20N cartridge in duplicate runs using a flow rate and temperature identical to those described above. The flow-through containing the anti-HPA-1a preparation was used to challenge Huh-7.5 cells, and the infectivity was compared to that of the spiked IgG preparation after 4 days of culture. Data (Fig 6) showed that nanofiltration efficiently and consistently removed HCV infectivity as assessed by luciferase reporter levels from an infection analysis using nanofiltered HCV-spiked IgG. Compared to the infectivity found in spiked IgG, HCV removal was over 3 log following nanofiltration and resulted in a baseline level of reporter activity found in the mock infection control. Thus, Planova 20N nanofiltration can efficiently remove HCV infectivity from anti-HPA-1a Ig.

## Discussion

Human plasma remains an important source of established or novel therapeutic products, including polyvalent and hyper-immune immunoglobulins like anti-D Ig [19], which are on the WHO model list of essential medicines [30]. Anti-HPA-1a IgG may prevent FNAIT based on AMIS, as found to be effective against HDN using anti-RhD IgG therapy [15], combined or not with non-invasive fetal RhD screening to identify RhD negative women carrying RhD positive fetuses [31]. Our main objective here was to provide a definite proof-of-concept of the feasibility of isolating anti-HPA-1a IgG from the plasma of alloimmunized women using a new manufacturing process approach meeting current requirements for product purity and viral safety [18]. Since HPA-1a plasma from alloimmunized women will likely be available in limited volumes, not compatible with the current scale of the traditional ethanol plasma fractionation industry (plasma pool of typically 4’000 liters) [18], a dedicated purification method is needed both for clinical proof-of-concept and future production. The starting plasma was obtained from women donors who were identified as having high level of anti-HPA-1a antibodies following an episode of alloimmunization during a previous pregnancy. MAIPA data at different time points in the four donors and in the plasma donations suggest a persistence of the antibodies in the circulation for more than 10 to 15 years. Collected plasma was subjected to cryoprecipitation to isolate the cryoprecipitate (a side-fraction which can be used to prepare factor VIII, Von Willebrand factor, and/or fibrinogen concentrate [18]). The cryoprecipitate-poor supernatant was treated by 5% caprylic acid at pH 5.5. We selected this treatment for three main reasons. First, caprylic acid is convenient, as it precipitates essentially all non-Ig proteins in a single step, yielding a supernatant containing not only a physiological proportion of all Ig classes and subclasses [22,32–34], but also anti-HPA-1a Igs, as revealed by PAK-12 and MAIPA data. Second, caprylic acid is already used to prepare therapeutic Igs, such as horse-plasma derived antivenoms [35,36], and human normal or hyperimmune Igs [21,37,38]. The CPP could be adsorbed onto DEAE-Sephadex A-50 resin prior to caprylic acid treatment to isolate prothrombin complex components, without affecting the Ig quality or yield, as already shown with polyvalent IgG [21]. Third, our caprylic acid treatment ensures robust inactivation of lipid-enveloped viruses [37,39,40], which is critical for designing virus safety margin in plasma product manufacturing processes. The Ig supernatant was subjected to S/D treatment with 1%TnBP/1%Triton X-100, a treatment that is known to ensure when performed at neutral to acidic pH an efficient inactivation of all lipid-enveloped plasma-borne viruses, such as the immunodeficiency virus, hepatitis B and HCV [41]. A two-steps chromatographic procedure combining S-HyperCel and HyperCel STAR AX [34] was successfully used to both remove the S/D agents and polish the anti-HPA-1a Ig fraction. The purified anti-HPA-1a Ig was readily nanofiltered on Planova 20N without changing the transmembrane pressure or decreasing the flow rate. Twenty-nanometer nanofiltration provides an additional viral safety margin against lipid-enveloped viruses, as shown by the removal of HCV, which was used as a relevant virus. The capacity of Planova 20N to remove non-enveloped viruses was not evaluated here but should be validated for clinical products under the processing conditions described in our study. Efficacy of 20-nm filters to remove small non-enveloped viruses, such as hepatitis A or parvovirus B19 is well established [42–44]. Planova 20N nanofiltration, as used here, is a recognized step for removal of non-enveloped viruses in IgG products [45,46]. Therefore the process includes three dedicated and complementary virus reduction steps to inactivate or remove all known plasma borne viruses, enveloped or non-enveloped, in full compliance with current European regulations [47]. Finally anti-HPA-1a Ig was concentrated to approximately 25~30 mg/ml. The anti-HPA-1a content, total protein content, IgG purity, non-detectable IgA presence in the final product are close to, or even exceeds, that of therapeutic anti-RhD preparations. Licensed anti-RhD product have an anti-D immunoglobulin content in the range of 625–750 IU/mL, a protein content close to 30 mg/mL, an IgG purity of 95–99%, an IgA content of 0.05% or less, and are complying with the European pharmacopoeia monograph for human anti-D immunoglobulins [48,49].

Analysis of fractions throughout the process by SDS-PAGE and protein zone electrophoresis confirmed that IgG was present, as expected, in CPP, the caprylic acid supernatant, S HyperCel Elution, HyperCel STAR AX breakthrough, Planova 20N filtrate, and after concentration and dialysis. The developed process yields IgG at a concentration of approximately 30 mg/mL, with high purity (>99.5%), and with low residual IgA and undetectable IgM, and largely free of other plasma proteins such as albumin and complement components C3 and C4. Due to increased thromboembolic complications found recently with therapeutic IgG [50,51], procoagulant factors were monitored. A TGA assay and chromogenic substrates were used to examine any signs of *in vitro* thrombogenicity [52]. The quantity of thrombin generated in the final anti-HPA-1a IgG was <350 nM, which is within current recommendations of regulatory authorities [52]. In addition, thrombin activity, proteolytic activity, and the plasmin level of purified IgG were essentially absent from the final product, which shows the capacity of the purification process to remove, or avoid the formation of, proteolytic enzymes, consistent with our previous studies [21]. The qualitative solid-phase ELISA (Pak12 kit) and quantitative MAIPA, that are used to detect or quantify anti-HPA-1a IgG in serum [53], were very useful tools to help refine purification of anti-HPA-1a, and indicated that it was co-purified with the bulk of IgG and concentrated in the final product. Interestingly, the anti-HPA-1a content in the final fraction by MAIPA was approximately 4~6-fold that of the CPP, which is comparable to the increment achieved in the total IgG content. Thus, the purification process did not lead to a specific loss of anti-HPA-1a IgG, nor its capacity to bind to HPA-1a antigen. Such procedure could likely be used for the preparation of IgG directed against other FNAIT antigenic platelet targets, such as HPA-2 on the GPIbα integrin, provided enough plasma can be obtained from alloimmunized donors. Another interesting possibility is that anti-HPA-1a plasma derived IgG preparations may also prevent immunization against other fetal platelet antigens, as well as contain high titers anti-alphaV beta-3 antibodies potentially capable to suppress corresponding alloimmunization. These antibodies were recently shown in an experimental murine model and in some human patients [54], to be responsible for impairment of vascular development and angiogenesis, and proposed to be the cause, possibly in conjunction with thrombocytopenia, of bleeding disorders and FNAIT-associated ICH. Future pre-clinical experiments will address the capacity of HPA-1a IgG in causing AMIS and finally being a candidate for prophylactic treatment to prevent immunization and thus FNAIT-induced bleeding complications.

In conclusion, our work shows the feasibility of fractionating anti-HPA-1a IgG from HPA-1a plasma using a scalable process meeting current requirements of quality and viral safety.
